# Construction of a preoperative scoring system to predict the difficulty level of colorectal endoscopic submucosal dissection

**DOI:** 10.1371/journal.pone.0219096

**Published:** 2019-06-27

**Authors:** Satohiro Matsumoto, Takeshi Uehara, Hirosato Mashima

**Affiliations:** Department of Gastroenterology, Saitama Medical Center, Jichi Medical University, Saitama, Japan; National Cancer Center, JAPAN

## Abstract

**Background:**

We attempted to examine the factors contributing to the difficulty in performance of colorectal ESD, with the aim of constructing a scoring system that could help in prediction of the difficulty level of the procedure.

**Methods and materials:**

The data were analyzed from two viewpoints: to determine the factors contributing to 1) non-*en bloc* resection and the factors contributing to 2) a slow resection speed. Factors falling under these two categories contributing to difficulty in performance of ESD were extracted and used to construct a scoring system. The validity of this scoring system was evaluated by calculating the correlation between the score and the resection speed in a different dataset.

**Results:**

Based on the results of our analysis, we assigned scores for various factors as follows: 4 points for EMR of a scarred lesion, 1 point for tumors with a diameter of ≥ 30 mm, 2 points for lesions located in the liver/splenic flexure, 1 point for lesions located in the transverse colon, 3 points for LST-NG-PD/depressed lesions, 1 point for protruded lesions and LST-NG-F lesions (range 0–10). In the validation study, the rank correlation coefficient between the score according to the scoring system and the resection speed was -0.130, representing a weak and negative correlation (P = 0.03). We defined the difficulty level depending on the sum of the scores: 0–2, low difficulty level; 3–5, intermediate difficulty level; ≥ 6, high difficulty level. The average resection speed was 12.6 mm^2^/min in the group with scores of 0–2, 8.1 mm^2^/min in the group with scores of 3–5, and 5.5 mm^2^/min in the group with scores of ≥ 6 (11.2 mm^2^/min in all lesions).

**Conclusion:**

Our colorectal ESD scoring system would be useful for selection of operators with the appropriate skill level in the procedure for colorectal ESD cases.

## Introduction

In Japan, colorectal endoscopic submucosal dissection (ESD) was approved for coverage by the national health insurance in April 2012, and this surgical procedure is now widely performed throughout the country [1–6]. Colorectal ESD is now considered as a standard treatment for early colorectal cancer, and a number of studies have reported its usefulness [2, 7–9]. However, colorectal ESD is technically more demanding than gastric ESD, and is associated with a higher incidence of procedural complications. Given that colorectal ESD is becoming popular in Japan, there is a need to develop a systematic method for training surgeons in performing ESD. It has been suggested that trainees should begin by performing colorectal ESD for laterally spreading tumor-granular type (LST-G) lesions measuring less than 40 mm in diameter [10]. Uraoka et al. recommend that beginners of colorectal ESD should begin with rectal LST-G lesions measuring 2–3 cm in diameter and then proceed to larger extra rectal LST-G lesions and thereafter to LST-non granular type (LST-NG) lesions measuring ≥ 2 cm in diameter [11].

At our hospital, endoscopists begin performing colorectal ESD after they have performed at least 20 cases of gastric ESD; however, recently, they have started to perform colorectal ESD for rectal LST-G lesions measuring 20–30 mm in diameter even before performing 20 gastric ESD cases. We agree that beginners of ESD for colorectal lesions should start with rectal lesions. However, such guidance alone may be insufficient, because there are other variables, such as the size and location of the tumors that should be taken into account when selecting operators with the appropriate level of skill for the procedure in individual patients. At teaching hospitals, like in our hospital, appropriate selection of lesions suitable for ESD beginners or trainees is often difficult.

In this study, we explored preoperative factors that could predict the difficulty level of colorectal ESD, with the aim of constructing a scoring system that would enable the treating doctor to predict the difficulty level of the procedure.

## Patients and methods

### Study population

**Fig 1:**
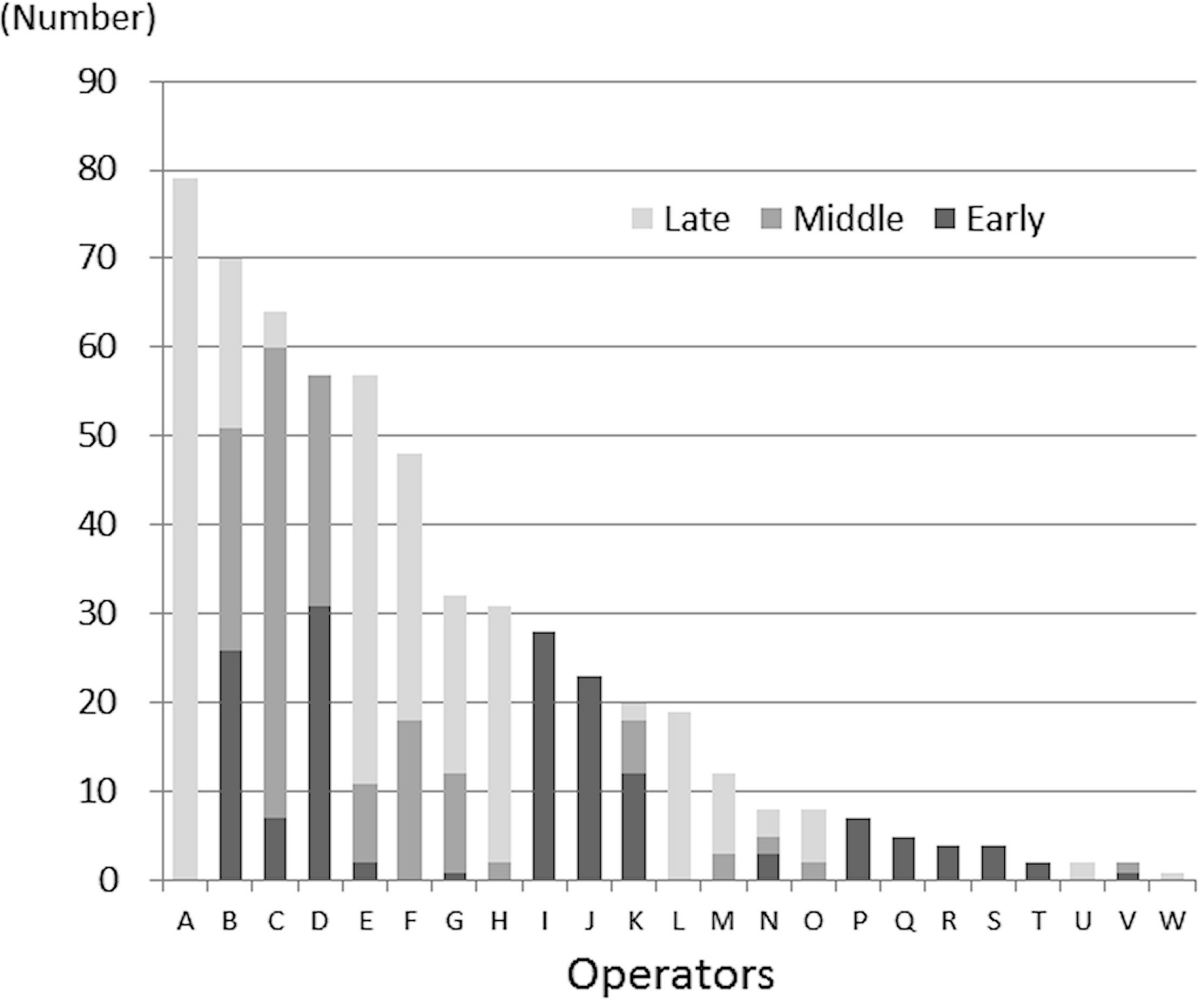
The numbers of colorectal ESD of each operator. Early, early period (2005–2010); Middle, middle period (2011–2013); Late, late period (2014–2015).

This was a retrospective single-center study conducted at the Saitama Medical Center. Data of a total of 549 patients with 583 colorectal tumors (excluding neuroendocrine tumors) who had undergone ESD at the Saitama Medical Center between January 2005 and December 2015 were enrolled. The clinical backgrounds (tumor size, location, macroscopic type, histology and experience of the operator) and the treatment outcomes (procedure time, *en bloc* resection rate, complete resection rate and complication rate), were analyzed in all the cases. The colorectal ESD procedures over the 11-year period had been carried out by a total of 23 operators with experience of having performed at least 20 cases of gastric ESD (Fig 1). Their experience in performing colorectal ESD was categorized according to the number of cases of colorectal ESD that they had performed, as follows: < 20, 20–49, and ≥50. Operators having experience of performing less than 20 colorectal ESDs were classified as beginner operators. In addition, the period after introduction of ESD in our hospital was divided according to the time period into the early period (2005–2010; 156 cases), middle period (2011–2013; 158 cases), and late period (2014–2015; 269 cases). All the data for this study were collected exclusively by reviewing the medical records of the patients. A subgroup analysis was performed of a restricted group of operators with experience of performing more than 20 colorectal ESDs during the study period. The subgroup included 10 operators and was considered as being more homogeneous in terms of the experience in performing ESD procedures (operator A to J, Fig 1).

### Setting of the difficulty of colorectal ESD

To evaluate the difficulty level in the performance of ESD, data were analyzed to identify factors contributing to 1) non-*en bloc* resection, and to 2) a slow resection speed. A multivariate analysis was performed using clinical background factors such as the tumor size, location, macroscopic type, histology, and experience of the operator or institution, to identify significant independent factors contributing to non-*en bl*oc resection, and the likelihood ratios of the identified factors were calculated. Then, the resection speed was calculated for each clinical background factor, and factors associated with a lower resection speed (lower than the mean) were extracted, and listed in order, from the slowest to the fastest. Subsequently, scores were assigned for all the extracted factors contributing to non-*en bloc* resection and low resection speed. The scoring for non-*en bloc* resection was based on the likelihood ratios, and that for the resection speed was based on weighting using the rank order and speed. Finally, a score sheet containing the two categories was developed, and the validity of this scoring system was evaluated by calculating the correlation between the score and the resection speed. The mean dissection speed for the resected lesions (mm^2^/min) was calculated to adjust the procedure time to the size of the resected lesion. The size of the resected lesion was directly measured after spreading it on a rubber plate. The area of the lesion was calculated using the formula to calculate the area of an ellipse (area = long axis×short axis×π/4). In cases of piecemeal resection in which the specimens could not be reconstructed, the lesion size was endoscopically determined.

### Validation of the scoring system

For the validation study, 177 patients with 183 colorectal tumors (excluding neuroendocrine tumors) who had undergone ESD between 2016 and 2017 were enrolled. The validity of this scoring system was evaluated by calculating the correlation between the score and the resection speed.

### ESD procedure

ESD was performed as previously reported elsewhere [12]. We followed the indications for colorectal ESD recommended by the colorectal ESD Standardization Implementation Working Group in Japan [7].

We usually use the GIF-Q260J (Olympus, Tokyo, Japan) endoscope for rectal lesions and the CF-Q260J or PCF-260J (Olympus) endoscope for lesions at other sites. Hyaluronic acid solution is essential as the submucosal injection solution; a 0.4% sodium hyaluronate solution for submucosal injection (Mucoup; Johnson & Johnson, Tokyo, Japan) is mixed in 10% glycerin containing 5% fructose and 0.9% NaCl (Glyceol; Chugai Pharmaceutical, Tokyo, Japan). A mucosal incision is made around the lesion and submucosal dissection for complete removal of the lesion is performed using the Flex knife (Olympus) or Dual knife (Olympus) or Flush knife (Fujinon, Tokyo, Japan). The Hook knife (Olympus) is also used, particularly in cases where dissection of the submucosa is difficult. As for the distal attachment, we use a short-type, Small-Caliber-Tip Transparent (ST) hood (Fujifilm Medical Co., Tokyo, Japan). A hemostatic forceps (Coagrasper, FD-410LR; Olympus Medical Systems Co., Tokyo, Japan) is used to control the bleeding during and after the procedure. A high-frequency generator (VIO 300D; ERBE, Tubingen, Germany) is used during incision of the mucosa. Traction devices were not used for any of the procedures conducted during the study period. We use carbon dioxide insufflation during the ESD. Intravenous cephem antibiotics are started on the day of the ESD and administered for approximately 3 days.

### Histopathological evaluation

All lesions are histopathologically examined on the basis of the Japanese criteria [13]. The macroscopic type is classified as the protruded type, depressed type, or laterally spreading tumor (LST). LSTs are formally defined as lesions larger than 10 mm in diameter, and subclassified into the granular type (LST-G) or non-granular type (LST-NG). LST-G lesions are further classified into the homogeneous type (LST-G-H) and nodular- mixed type (LST-G-M) lesions, while LST-NG lesions are further classified into the flat-elevated type (LST-NG-F) and pseudo-depressed type (LST-NG-PD) lesions. Lesions are also classified according to the depth of invasion of the submucosal layer (SM) as SM1 lesions (< 1000 μm from the muscularis mucosae) and SM2 lesions (≥ 1000 μm submucosal invasion). Fibrosis of the submucosal layer is classified into 3 grades (F0-2) according to the appearance of the layers during submucosal injection of the mixture of sodium hyaluronate and indigo carmine, as described previously [14]. The tumors are classified as adenoma, differentiated cancer or sessile serrated adenoma/polyp (SSA/P).

*En bloc* resection is defined as resection in a single piece. Complete (R0) resection is defined as *en bloc* resection of a tumor with a negative horizontal margin and vertical margin. Curative resection is defined as *en bloc* R0 resection of a tumor without SM2 invasion or lymphovascular invasion.

### Adverse events

Both perioperative and postoperative perforations are counted as perforation. Postoperative bleeding is defined as fresh gross hematochezia after the procedure, requiring colonoscopy.

### Ethics statement

This study was conducted with the approval of the Etiological Study Ethical Review Board of Saitama Medical Center, Jichi Medical University (S14-132). Because we produced anonymized data for use in this study, it was deemed not necessary to obtain informed consent from the study subjects.

### Statistical analysis

Data are expressed as means ± SD or percentages. Statistical analysis was performed using the Student’s *t*-test and Fisher’s exact test. The macroscopic type, histology, and grade of fibrosis were compared by the chi-square test. One-way analysis of variance (ANOVA) with post hoc Tukey–Kramer tests was used to analyze the differences among the three groups. Factors identified as being significant by univariate analysis (P<0.15) were entered into the multivariate logistic regression analysis model. The Spearman's rank correlation coefficient was used to measure the strength and direction of the correlation between two variables. All the data analyses were performed using the StatView software (version 5.0; SAS Institute Inc., Cary, NC, USA). Differences at *P* values of less than 0.05 were regarded as being significant.

## Results

### Clinical characteristics and outcomes of ESD

**Fig 2:**
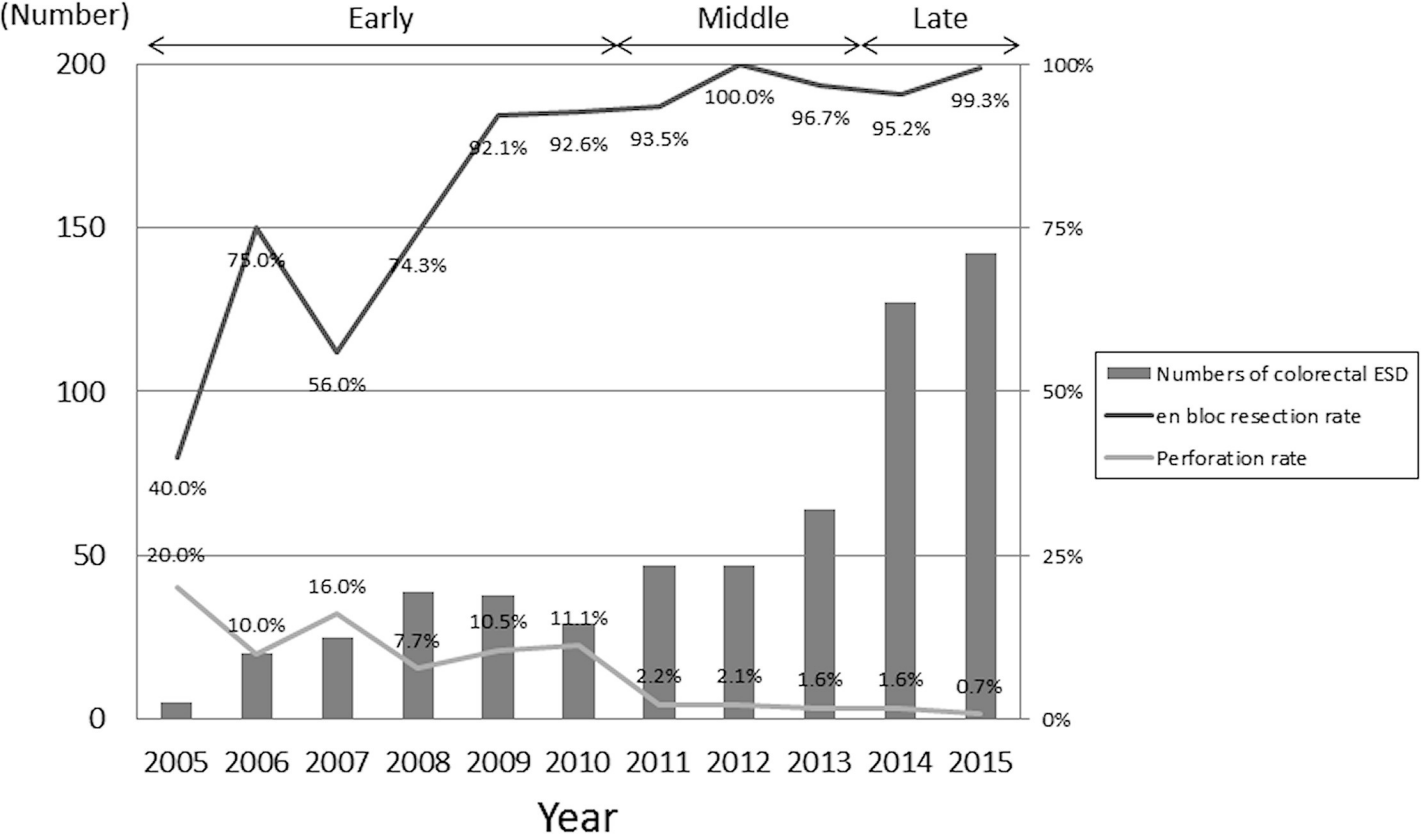
The differences in the numbers of colorectal ESDs, *en bloc* resection rate, and perforation rate.

The subjects comprised 339 men and 224 women, with a mean age of 68 ± 9 years. The mean tumor diameter was 28.9 ± 15.3 mm. The most frequent lesion location was the rectum (166 lesions; 28.5%), followed by the ascending colon (117 lesions; 20.1%) and transverse colon (100 lesions; 17.2%). The macroscopic type was classified as the protruded type (67 lesions; 11.5%), depressed type (8 lesions; 1.4%), LST-G (245 lesions; 42.0%; these lesions included LST-G-H lesions in 133 cases and LST-G-M lesions in 112 cases), or LST-NG (263 lesions; 45.1%; these lesions included LST-NG-F lesions in 149 cases and LST-NG-PD lesions in 114 cases). In regard to the severity of fibrosis, 166 lesions (28.5%) were classified as F1 and 35 lesions (6.0%) as F2, among which an endoscopic mucosal resection (EMR) scar lesion was found in 5 cases (0.9%). The numbers of lesions resected classified according to the colorectal ESD experience of the operators were as follows: operators with experience of < 20 cases, 214 lesions (36.7%); operators with experience of ≥ 20, but < 50 cases, 290 lesions (49.7%); operators with experience of ≥ 50 cases, 79 lesions (13.6%). The mean procedure time was 79.4 ± 64.7 min. The *en bloc* resection rate was 91.3%, and the curative R0 resection rate was 81.6%. Perforation was found in 24 patients (4.1%), of which 4 patients required emergency surgery, although the postoperative course was uneventful in all the 4 patients. Postoperative bleeding was found in 17 patients (2.9%) (Table 1). Fig 2 shows the differences in the numbers of colorectal ESDs, *en bloc* resection rate, and perforation rate according to the period. The *en bloc* resection rate and perforation rate in the early period (2005–2010) were 77.6% and 11.5%, respectively, those in the middle period (2011–2013) were 94.9% and 1.9%, respectively, and those in the late period (2014–2015) were 97.0% and 1.1%, respectively, showing an improvement of both the *en bloc* resection rate and perforation rate with time.

**Table 1:** Patient and lesion characteristics, and outcomes.

	Total (n = 583)	Subgroup* (n = 486)
Age, year, mean ± SD (range)	68 ± 9 (35–89)	68 ± 9 (35–89)
Male / female	339 / 244	280 / 206
Location		
Cecum	55 (9.4%)	47 (9.7%)
Ascending	117 (20.1%)	98 (20.2%)
Hepatic flexure	29 (5.0%)	28 (5.8%)
Transverse	100 (17.2%)	77 (15.8%)
Splenic flexure	14 (2.4%)	12 (2.5%)
Descending	24 (4.1%)	14 (2.9%)
Sigmoid	97 (16.6%)	78 (16.4%)
Rectum	166 (28.5%)	133 (27.4%)
Size, mm, mean ± SD (range)	28.9 ± 15.3 (3–112)	32.5 ± 14.6 (3–112)
Macroscopic type		
Protruded	67 (11.5%)	53 (10.9%)
Depressed	8 (1.4%)	7 (0.2%)
LST-G	245 (42.0%)	205 (42.2%)
LST-G-H / -M	133 / 112	106 / 99
LST-NG	263 (45.1%)	221 (45.5%)
LST-NG-F / -PD	149 / 114	119 / 102
Histology		
Adenocarcinoma	378 (64.8%)	313 (64.4%)
Intramucosa	282 (48.4%)	233 (47.9%)
SM1	47 (8.1%)	41 (8.4%)
SM2	49 (8.4%)	40 (8.2%)
Adenoma	173 (29.7%)	143 (29.4%)
SSA/P	32 (5.5%)	30 (6.2%)
Fibrosis	201 (34.5%)	168 (34.6%)
F1/ F2	166 / 35	143 / 25
Procedure time, min, mean ± SD (range)	79.4±64.7 (10–600)	76.2 ±64.1 (10–600)
*en bloc* resection rate, %	91.3%	93.6%
Curative resection rate, %	81.6%	83.7%
Perforation	24 (4.1%)	16 (3.3%)
Postoperative bleeding	17 (2.9%)	13 (2.7%)
Emergency surgery	4 (0.7%)	3 (0.6%)
Additional surgery	51 (8.7%)	41 (8.4%)
Operator experience performing CRESD		
<20 ESDs	214 (36.7%)	198 (40.7%)
21–49 ESDs	290 (49.7%)	209 (43.0%)
≥50 ESDs	79 (13.6%)	79 (16.3%)

*Subgroup included 10 operators having experience of performing more than 20 colorectal ESD during the study period. LST, laterally spreading tumor; LST-G, granular type; LST-NG, non-granular type; LST-G-H, homogenous type; LST-G-M, nodular mixed type; LST-NG-F, flat elevated type; LST-NG-PD, pseudo depressed type; SM1, < 1000 μm from the muscularis mucosae; SM2, ≥ 1000 μm submucosal invasion; SSA/P, sessile serrated adenoma/polyp; CR, colorectal

### Analysis of factors contributing to non-en bloc resection

Univariate analysis of the clinical background factors, including tumor factors, showed that the significant risk factors for non-*en bloc* resection were: colorectal ESD in the early period (OR, 7.43; 95% CI, 3.98–13.89), lesion diameter ≥ 30 mm (OR, 2.13; 95% CI, 1.19–3.84), and colorectal ESD performed by operators with experience of < 20 cases (OR, 5.91; 95% CI, 1.39–2.50). Multivariate analysis identified colorectal ESD in the early period (OR, 5.37; 95% CI, 2.70–10.68), lesion diameter ≥ 30 mm (OR, 2.50; 95% CI, 1.29–4.86), and depressed (LST-NG-PD/depressed) lesions (OR, 2.50; 95% CI, 1.23–5.06) as independent risk factors. The positive likelihood ratios of tumor-related factors, i.e., lesion diameter ≥ 30 mm and depressed lesions were 1.45 (95% CI, 1.04–2.03) and 1.16 (95% CI, 0.96–1.41), respectively (Table 2).

**Table 2:** Preoperative indicators for failure of *en bloc* resection (n = 583).

	Failure of *en bloc* resection			
	Univariate analysis		Multivariate analysis	
	OR (95% CI)	*P* value	OR (95% CI)	*P* value
Location		0.60		
Rectum	1			
Flexure	1.07 (0.33–3.41)			
Cecum	1.05 (0.36–3.02)			
Ascending	0.64 (0.24–1.73)			
Transverse	1.16 (0.50–2.70)			
Descending	0.60 (0.15–2.30)			
Sigmoid	0.94 (0.38–2.31)			
Tumor size, mm		0.01		0.006
<30	1		1	
≥30	2.13 (1.19–3.84)		2.50 (1.29–4.86)	
Depressed lesion (LST-NGPD/depressed)		0.07		0.01
No	1		1	
Yes	1.81 (0.98–3.36)		2.50 (1.23–5.06)	
Operator experience of CRESD		0.0004		0.07
≥20	1		1	
<20	5.91 (1.39–2.50)		1.97 (0.93–4.19)	
Initial period after the introduction of CRESD		0.01		<0.0001
No	1		1	
Yes	7.43 (3.98–13.89)		5.37 (2.70–10.68)	

LST, laterally spreading tumor; LST-NG, non-granular type; LST-NG-PD, pseudo depressed type; CR, colorectal

#### Analysis of factors contributing to a slow resection speed

The mean resection speed was 11.31 mm^2^/min. Table 3 shows the factors associated with a lower resection speed. The resection speed was the slowest for EMR scar lesions (4.13 mm^2^/min), followed by depressed (LST-NG-PD/depressed) lesions (7.84 mm^2^/min), lesions located in the colonic flexures (hepatic flexure and splenic flexure) (8.18 mm^2^/min), LST-NG-F lesions (9.69 mm^2^/min), and lesions located in the transverse colon (9.92 mm^2^/min) (Table 3).

**Table 3:** Resection speed in lesion characteristics and operator experience (mm^2^/min).

	Total (n = 583)	*P* value	Subgroup* (n = 486)	*P* value
Lesion in whole	11.31 ± 7.50		11.71 ± 7.45	
Location		0.002		0.0008
Cecum	13.81 ± 9.62		14.56 ± 9.74	
Ascending	12.52 ± 6.45		12.72 ± 6.50	
Transverse	9.92 ± 6.96		10.13 ± 6.18	
Descending	11.67 ± 11.01		14.31 ± 11.15	
Sigmoid	10.91 ± 7.16		11.04 ± 7.36	
Rectum	11.59 ± 7.52		12.02 ± 7.52	
Flexure (hepatic/splenic)	8.18 ± 5.26		8.38 ± 5.33	
Macroscopic type		<0.0001		0.0003
Protruded	10.04 ± 7.10		10.78 ± 7.22	
Depressed	7.39 ± 10.09		8.35 ± 10.49	
LST-GH	14.19 ± 8.31		14.31 ± 8.40	
LST-GM	14.53 ± 7.91		14.64 ± 7.74	
LST-NGF	9.69 ± 6.06		10.24 ± 6.06	
LST-NGPD	7.87 ± 5.12		8.34 ± 5.33	
Depressed lesion (LST-NGPD/depressed)	7.84 ± 5.51		8.34 ± 5.73	
EMR scar	4.13 ± 2.32		4.81 ± 2.11	
Operator experience performing CRESD		<0.0001		<0.0001
<20 ESDs	9.82 ± 7.20		10.11 ± 7.07	
21–49 ESDs	12.66 ± 7.71		12.67 ± 7.73	
≥50 ESDs	13.19 ± 7.00		13.19 ± 7.00	

*Subgroup included 10 operators having experience of performing more than 20 colorectal ESD during the study period. LST, laterally spreading tumor; LST-G, granular type; LST-NG, non-granular type; LST-G-H, homogenous type; LST-G-M, nodular mixed type; LST-NG-F, flat elevated type; LST-NG-PD, pseudo depressed type

### Subgroup analysis of factors contributing to non-en bloc resection and slow resection

Multivariate analysis identified colorectal ESD in the early period (OR, 4.90; 95% CI, 2.12–11.31) and depressed (LST-NG-PD/depressed) lesions (OR, 2.52; 95% CI, 1.09–5.78) as being independent risk factors for non-*en bloc* resection (Table 4). The mean resection speed was 11.71 mm^2^/min. Table 3 shows the factors associated with a lower resection speed. The resection speed was the slowest for EMR scar lesions (4.81 mm^2^/min), followed by depressed (LST-NG-PD/depressed) lesions (8.34 mm^2^/min) and lesions located in the colonic flexures (hepatic flexure and splenic flexure) (8.38 mm^2^/min) (Table 3). The results of the subgroup analysis showed the same trend as the original analysis.

**Table 4:** Preoperative indicators for failure of *en bloc* resection in the subgroup (n = 486).

	Failure of *en bloc* resection			
	Univariate analysis		Multivariate analysis	
	OR (95% CI)	*P* value	OR (95% CI)	*P* value
Location		0.97		
Rectum	1			
Flexure	0.90 (0.28–3.57)			
Cecum	1.10 (0.28–4.33)			
Ascending	1.15 (0.38–3.43)			
Transverse	0.89 (0.29–2.66)			
Descending	0.97 (0.11–8.40)			
Sigmoid	0.90 (0.23–3.57)			
Tumor size, mm		0.13		0.08
<30	1		1	
≥30	1.74 (0.84–3.62)		2.00 (0.91–4.41)	
Depressed shaped (LST-NGPD/depressed)		0.07		0.02
No	1		1	
Yes	2.00 (0.93–4.32)		2.52 (1.09–5.78)	
Operator experience of CRESD		0.02		0.44
≥20	1		1	
<20	2.44 (1.16–5.15)		1.40 (0.60–3.26)	
Initial period after the introduction of CRESD		<0.0001		0.0002
No	1		1	
Yes	5.69 (2.69–12.06)		4.90 (2.12–11.31)	

LST, laterally spreading tumor; LST-NG, non-granular type; LST-NG-PD, pseudo depressed type; CR, colorectal

### Scoring system for predicting the difficulty level of colorectal ESDs

Lesion diameter ≥ 30 mm and depressed (LST-NG-PD/depressed) lesions were assigned a score of 1 point each, because the positive likelihood ratios of the two risk factors for non-*en bloc* resection were not high. In regard to the resection speed, EMR scar lesions were assigned a score of 4 points, depressed (LST-NG-PD/depressed) lesions and lesions in the colonic flexures were assigned a score of 2 points each, lesions in the transverse colon, elevated-type and LST-NG-F lesions were assigned a score of 1 point each, and a scoring system with a total score range of 0 to 10 was created (Table 5).

**Table 5:** Scoring system on difficulty of colorectal ESD.

	Failure of *en bloc* resection	Resection speed	Total score
EMR scar	0	4	4
Tumor size ≥30mm	1	0	1
Flexure (hepatic/splenic)	0	2	2
Transverse	0	1	1
Depressed lesion (LST-NGPD/depressed)	1	2	3
Protruded	0	1	1
LST-NG-F	0	1	1

LST, laterally spreading tumor; LST-NG, non-granular type; LST-NG-F, flat elevated type; LST-NG-PD, pseudo depressed type; CR, colorectal

### Evaluation of the scoring system for predicting the difficulty level of colorectal ESDs based on analysis of the resection speed

**Fig 3:**
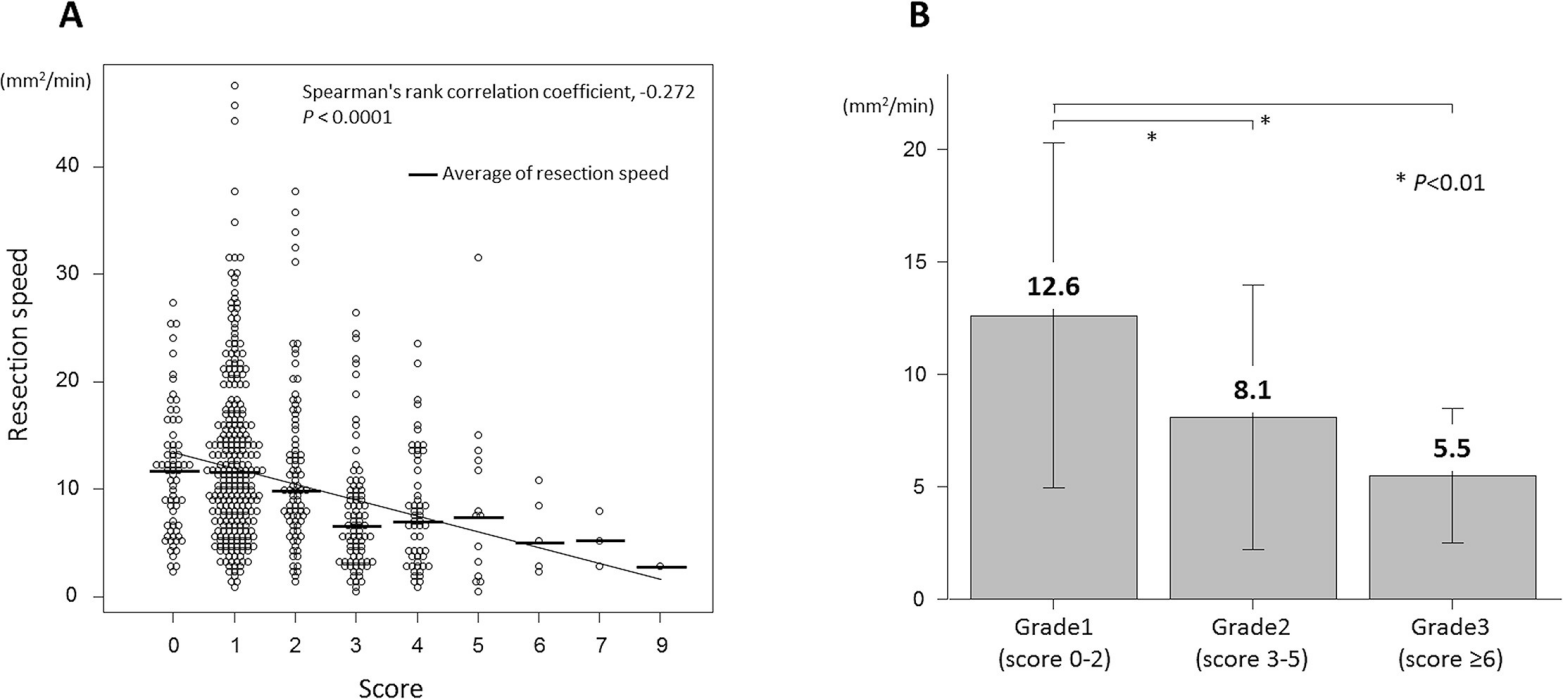
Association between the score and the resection speed. (A) The rank correlation coefficient between the score according to the scoring system and the resection speed. (B) The mean resection speed for three grades.

The rank correlation coefficient between the score according to the scoring system and the resection speed was -0.272, denoting a weak and negative correlation (P < 0.0001). Then, the scores were divided into three grades (Grade 1, 0–2 points; Grade 2, 3–5 points; Grade 3, ≥ 6 points), and the mean resection speed for each group was calculated as 12.6 ± 7.7 mm^2^/min for Grade 1 patients, 8.1 ± 5.9 mm^2^/min for Grade 2 patients, and 5.5 ± 3.0 mm^2^/min for Grade 3 patients. There were significant differences between Grade 1 and Grade 2 patients and also between Grade 1 and Grade 3 patients (P < 0.01) (Fig 3A and 3B).

### Validation of the scoring system for predicting the difficulty level of colorectal ESDs based on analysis of the resection speed

**Fig 4:**
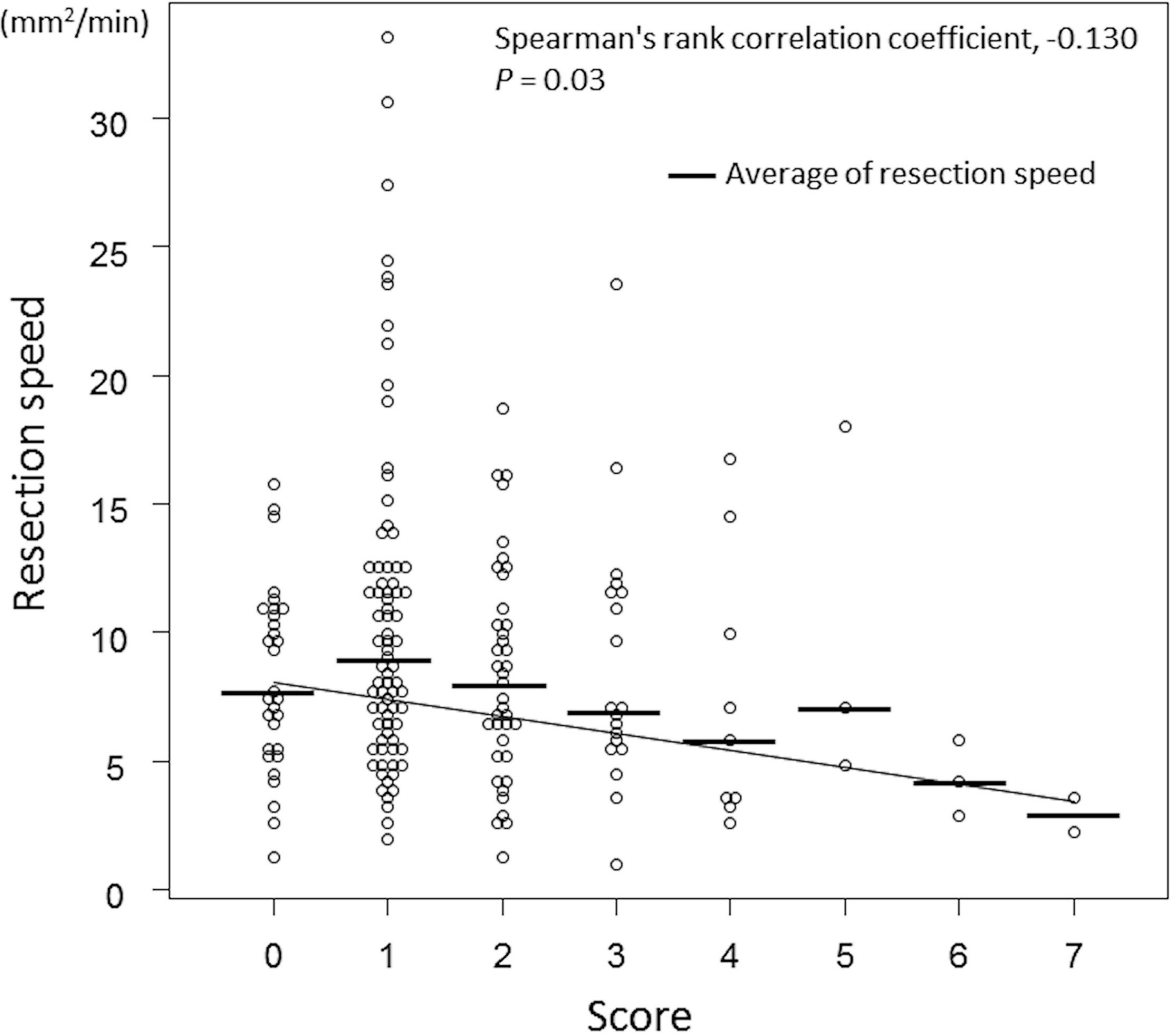
Validation of the scoring system for predicting the difficulty level of colorectal ESDs. The rank correlation coefficient between the score according to the scoring system and the resection speed was evaluated using the dataset of colorectal ESD cases that was performed in 2016 and 2017.

We assigned scores (1–4) according to the lesion characteristics and the location, which might seem arbitrary. Therefore, we also performed a validation study, using the data of colorectal ESDs performed in 2016 and 2017. The rank correlation coefficient between the score according to the scoring system and the resection speed was -0.130, representing a weak and negative correlation (P = 0.03) (Fig 4).

## Discussion

There are few previous reports of a scoring system for predicting the difficulty level of ESD for gastrointestinal lesions, which prompted us to perform this study. It has been reported that trainees reach a stable level of performing the procedure safely and independently after gaining experience of 20–30 cases of colorectal ESD [15, 16]. On the other hand, one study reported that trainees required experience of at least 80 cases of colorectal ESD to acquire the appropriate level of skill in performing the procedure [17]. In addition, it has been recommended that trainees should perform at least 30 cases of gastric ESD before performing colorectal ESD, and start with rectal LST-G lesions measuring approximately 30 mm in diameter and then proceed gradually to smaller lesions in the distal colon and larger lesions in the proximal colon [18]. A study on the learning curve of an endoscopist for colorectal ESD showed that the number of cases required to reach an *en-bloc* resection rate of 80% was 5 cases for rectal ESD and 20 cases for colon ESD, indicating that it takes more time to acquire the skill for colon ESD as compared to that for rectal ESD. Thus, the learning curve for colorectal ESD is different from that for rectal ESD [19]. A study in Japan examined the learning curve for colorectal ESD in clinical practice by providing training in colorectal ESD using 12 lesions in pig models to two trainees with no experience in performing gastric ESD. They reported that the *en bloc* resection rate in the first 20 cases for both trainees was 100%, and the perforation rates in the first 20 cases were 0% and 10%, respectively, showing favorable results, including in terms of the complication rates [20]. Similarly, other studies have reported that training using animal models shortened the learning curve for ESD in clinical practice [21, 22]. On the other hand, a study in Korea examining the learning curve for colorectal ESD in endoscopists with no experience in gastric ESD or training using animal models reported that the *en bloc* resection rate and peroration rate in the first 150 cases were 90% and < 10%, respectively [23]. These findings suggest that prior learning, such as training using animal models, is quite important for trainees with no experience in gastric ESD or those with less experience. Early gastric cancer cases indicated for ESD are few in Western countries; therefore, it is recommended that trainees from western countries gain practice using an *in vitro* porcine training model [24].

In our study, multivariate analysis identified tumor diameter ≥ 30 mm and depressed (LST-NG-PD/depressed) lesions as significant risk factors for non-*en bloc* resection. Fold convergence, underlying semilunar folds, and protruding-type lesions have also been reported as risk factors for non-*en bloc* resection and perforation in patients undergoing colorectal ESD [25]. Fibrosis of the submucosal layer has been reported as a risk factor for difficulty in performing ESD [26], piecemeal resection and perforation [27]. Furthermore, severe fibrosis of the submucosal layer (F2) was associated with a slow resection speed [28] and a lower *en bloc* resection rate, which did not improve with the learning curve [14]. Thus, predicting the presence of fibrosis is extremely important in preoperative diagnosis. The reported risk factors for severe fibrosis (F2) are a tumor diameter of ≥ 30 mm (OR, 3.16; 95% CI, 1.52–6.56) and LST-NG-PD-type lesions (OR, 3.61; 95% CI, 0.98–13.35) [29].

In this study, resection speed was used as a parameter for predicting the difficulty level of colorectal ESD. In particular, EMR scar lesions and depressed (LST-NG-PD/depressed) lesions were associated with a lower resection speed; and the larger the tumor size, the higher the resection speed. The difficulty level of ESD was higher for scar lesions in the colon and rectum. Furthermore, ESD was also more difficult for LST-NG and sessile-type lesions in the colon, but not the rectum [28]. A study examined the difficulty of ESD in the rectum and sigmoid colon by defining difficulty as a resection speed of ≥ 120 minutes or piecemeal resection, and reported that the tumor size was associated with procedural difficulty and that there was a strong positive correlation between the tumor size and the procedure time [27]. In a study on the risk factors for colorectal ESD requiring 2 hours or longer, multivariate analysis identified a tumor diameter of ≥ 4 cm [30, 31], less endoscopist or institution experience in ESD [30], and paradoxical movements of the endoscope [31] were significant risk factors. In another study, longer procedure time was defined as a resection time of ≥ 150 minutes, based on the findings that the mean operative time for laparoscopic surgery was 165 minutes [32] or 156 minutes [33]. The authors reported that lesion location at the colonic flexures, ileocecal valve or dentate line area, a tumor diameter of ≥ 50 mm, scar lesions, and LST-NG-type lesions were significant risk factors for a longer procedure time [34].

Perforation is an important complication of colorectal ESD. The reasons for the relatively higher incidence of perforation with colorectal ESD include the narrow intestinal tract, the thin intestinal wall, and the long and curved intestinal tract. Fibrosis of the submucosal layer [14, 29, 35] and lesion location at flexures [34] were reported as risk factors for perforation complicating colorectal ESD. Hong et al. performed a multivariate analysis of risk factors for perforation associated with colorectal ESD, reporting that ESD experience of ≥ 50 cases of ESD (OR, 0.59), each 1 cm increase in tumor diameter (OR, 1.39), lesion location in the colon outside the rectum (OR, 2.20), and fibrosis of the submucosal layer (OR, 2.00) were significant risk factors. Similar results were obtained in a validation study. In the study by Hong et al., scoring was performed on four items as follows: experience of ≥ 50 cases of ESD was assigned a score of -1, each 1 cm increase in tumor diameter was assigned a score of +1 per 1cm, tumor location in the colon outside the rectum was assigned a score of +2, and fibrosis of the submucosal layer was assigned a score of +2; then, patients with a total score of ≤ 4 points were classified as the low-risk group, and those with a total score of >4 were classified as the high-risk group [36].

We believe that the preoperative scoring system for predicting the difficulty level of colorectal ESDs created in this study would enable appropriate selection of operators according to the difficulty level of the procedure, and contribute to reducing the procedure time, improving the *en bloc* resection rate, and reducing the incidence of complications. When using this scoring system, we recommend that Grade 1 patients be assigned to endoscopists with experience of < 20 cases, Grade 2 patients to endoscopists with experience of ≥ 20 cases, and Grade 3 patients to endoscopists with experience of ≥ 50 cases of colorectal ESD.

The limitations of this study include its retrospective nature and single-center design. Although the operability of the endoscope is an important factor associated with non-*en bloc* resection [25, 31], information about this during the early period was insufficient; therefore, it was excluded from the analysis. Experience of performing at least 20–30 cases of gastric ESD is necessary before a trainee can start performing colorectal ESD [15, 17, 18]. In our experience, at least 20 cases of gastric ESD should be completed before a trainee begins to perform colorectal ESD. However, in this study, experience in performing ESD was evaluated only based on the experience of colorectal ESD, without considering the experience in performing gastric ESD. In addition, fibrosis was finally excluded from the potentially associated factors, since accurate assessment of fibrosis is not possible without performing dissection of the submucosal layer and therefore preoperative diagnosis of fibrosis is impossible.

## Conclusion

Colorectal ESD is technically challenging; therefore, careful selection of operators according to an objective standard is important. We believe that our colorectal ESD scoring system would enable appropriate selection of operators based on the learning curve.
